# Probabilistic Attention Map: A Probabilistic Attention Mechanism for Convolutional Neural Networks

**DOI:** 10.3390/s24248187

**Published:** 2024-12-22

**Authors:** Yifeng Liu, Jing Tian

**Affiliations:** NUS-ISS, National University of Singapore, Singapore 119615, Singapore; liu.yifeng@u.nus.edu

**Keywords:** attention mechanism, convolutional neural networks, probabilistic attention

## Abstract

The attention mechanism is essential to *convolutional neural network* (CNN) vision backbones used for sensing and imaging systems. Conventional attention modules are designed heuristically, relying heavily on empirical tuning. To tackle the challenge of designing attention mechanisms, this paper proposes a novel probabilistic attention mechanism. The key idea is to estimate the probabilistic distribution of activation maps within CNNs and construct probabilistic attention maps based on the correlation between attention weights and the estimated probabilistic distribution. The proposed approach consists of two main components: (i) the calculation of the probabilistic attention map and (ii) its integration into existing CNN architectures. In the first stage, the activation values generated at each CNN layer are modeled by using a Laplace distribution, which assigns probability values to each activation, representing its relative importance. Next, the probabilistic attention map is applied to the feature maps via element-wise multiplication and is seamlessly integrated as a plug-and-play module into existing CNN architectures. The experimental results show that the proposed probabilistic attention mechanism effectively boosts image classification accuracy performance across various CNN backbone models, outperforming both baseline and other attention mechanisms.

## 1. Introduction

Image analysis and pattern recognition are critical to the fields of sensing and imaging, particularly in applications where camera sensors are used to capture and process color images. Modern imaging systems typically generate a vast number of visual data that need to be analyzed efficiently and accurately in computer vision tasks, such as autonomous vehicles, medical diagnostics, and surveillance.

In recent years, *convolutional neural networks* (CNNs) trained on large-scale image datasets have revolutionized computer vision tasks [1], achieving great success in areas such as image classification [2] and enhancement [3]. CNNs owe their effectiveness to two key advantages. First, they excel at learning hierarchical feature representations, allowing models to capture complex patterns in images through a set of layers of convolutional filters. These filters progressively extract low-level details such as edges and textures, which are then combined into more abstract, high-level features. Second, CNNs benefit from a vast number of labeled data and optimization techniques, allowing models to generalize well to unseen data after being trained on extensive image datasets like ImageNet [4]. These advantages have positioned CNNs as the popular backbones of modern vision-based imaging and sensing systems.

This paper focuses on how to further improve the performance of CNNs in the context of image recognition for imaging and sensing systems. For camera-based sensing systems, this enhancement can lead to the more accurate detection of critical features in complex environments [5,6], such as identifying subtle patterns in medical imaging or recognizing objects under low-light conditions in autonomous driving systems.

Rather than redesigning entire CNN architectures from scratch, many recent research works have focused on improving existing architectures by developing advanced components to boost performance. One of the most influential ideas is the introduction of attention mechanisms [7,8]. Inspired by the human visual system, which selectively focuses on the most important parts of a scene, attention mechanisms enable CNNs to dynamically emphasize the most relevant regions or features in an image. By selectively weighting features based on their importance, attention modules allow networks to process more meaningful information while suppressing irrelevant details, thus improving performance in a variety of vision tasks, such as object detection [9], text-to-image generation [10,11], and video classification [12]. When incorporated into CNNs, attention mechanisms function as a dynamic selection process, focusing on different parts of the input image depending on the task at hand. This process is similar to how humans focus on particular regions of a scene while ignoring less relevant background information. It augments the feature extraction process by applying varying weights to different features, thereby enhancing the model’s ability to capture critical aspects of the input data. By integrating this attention mechanism into existing CNN architectures, this study provides a scalable and flexible solution that can be adapted to various sensing platforms. Furthermore, the seamless integration into CNNs ensures that this method can be applied without requiring major redesigns of existing systems, making it an attractive option for industries seeking to upgrade their imaging capabilities. For example, in the context of medical imaging, where precise image analysis is critical for diagnosis and treatment, attention mechanisms could enhance the performance of CNNs used in identifying and classifying medical conditions from modalities such as MRI or X-rays. For example, in tumor detection or anomaly identification, this attention mechanism would allow CNNs to focus more effectively on subtle, critical regions of the image that might be overlooked by conventional attention methods. In addition, the mechanism can be extended to applications in autonomous systems, such as self-driving cars, where real-time image analysis is crucial for object detection and scene understanding. The attention map could help the vehicle vision system prioritize important visual signals, such as pedestrians or road signs.

However, conventional attention modules are designed heuristically and lack a strong theoretical foundation. They often rely on empirical experimentation to fine-tune their structure and operations. The absence of a solid interpretative framework for attention mechanisms has made it challenging to systematically improve them, as their design typically involves a trial-and-error process rather than being guided by principled reasoning.

To address this challenge, this paper proposes a novel probabilistic approach to designing attention mechanisms. The contributions of this paper are twofold:

- Instead of relying on heuristic methods, the proposed approach estimates the probabilistic distribution of activation maps within the CNN and constructs an attention map based on the correlation between attention weights and the estimated probability density function values. The experimental results verify that the proposed Laplace distribution can fit the activation map distribution more accurately than other distributions.
- The proposed probabilistic attention map, informed by the distribution of activations, is incorporated as a plug-and-play module into existing CNN architectures to improve their performance in image classification tasks. The experimental results demonstrate that the proposed method boosts image classification accuracy.

The rest of this paper is organized as follows: Section 2 introduces the relevant works on attention mechanisms. The proposed probabilistic attention mechanism is presented in Section 3, including a formulation of the distribution of activations and a plug-and-play integration strategy. Then, the proposed approach is evaluated in an image classification task in Section 4 with an ablation study. Finally, Section 5 concludes this paper.

## 2. Related Works

This section provides an overview of various types of attention mechanisms used in CNNs [7,8]. These representative works can be categorized into the following aspects, as summarized in Table 1: channel attention, non-local attention, self-attention, and spatial attention. Additionally, this section highlights recent works that explore the usage of probabilistic information to refine attention mechanisms.

### 2.1. Conventional Attention Mechanisms

The first category includes the channel attention mechanisms [13,14,15,16,17], which focus on identifying and emphasizing the most informative feature channels within a CNN model. Typically, a CNN model processes images through multiple layers, generating a series of feature maps, each containing different channels. Not all channels carry equal importance; some channels may contain more relevant information about the object or scene being processed, while others may represent noise or redundant features. Based on this observation, channel attention selectively weights these channels based on their significance, allowing the CNN model to amplify important channels and suppress less relevant ones. For example, the *squeeze-and-excitation* (SE) block, which is proposed in [13], introduces a global pooling operation followed by a two-layer fully connected network to learn the importance of each channel.

The second category is non-local attention [18], which focuses on capturing long-range dependencies within feature maps. Unlike traditional convolutional layers, which apply a local filter to a small region of the image, non-local attention considers the relationships between all pixel locations in the feature map. Due to its ability to capture global dependencies, it allows the CNN model to gather rich contextual information, which is particularly useful in tasks that require understanding large-scale structures. More specifically, in this attention mechanism, the similarity between two distant pixels is computed, and attention is applied based on this similarity. By attending to distant yet related pixels, the model can better recognize relationships between different parts of the input.

The third category is self-attention [19], which is a mechanism initially popularized by the transformer architecture [29]. Self-attention calculates attention scores for each element (or pixel) in the input sequence (or feature map) relative to all other elements. This allows the model to capture dependencies across the entire input image. In sensing and imaging, self-attention allows the model to process images as sequences of patches, capturing global relationships between different parts of the image. This is very different from traditional CNNs, which rely on local receptive fields.

The fourth category is spatial attention [20,21], which directs the CNN model’s focus to specific regions within the spatial domain of the image. Unlike channel attention, which operates along the channel dimension, spatial attention focuses on the spatial arrangement of the pixels in the feature map. It assigns different weights to different spatial locations, highlighting the most informative regions and suppressing irrelevant areas. Spatial attention is typically implemented by applying a 2D convolution over the feature map to generate an attention map, which is then used to re-weight the feature map spatially.

It is important to note that several works [22,23,24,25] have combined channel attention and spatial attention to create a more comprehensive attention mechanism. These approaches allow the CNNs to attend to both important channels and key spatial regions simultaneously. For example, the *Convolutional Block Attention Module* (CBAM) [22] first applies channel attention to focus on important features and then applies spatial attention to highlight the relevant regions in the feature map. *Dense-and-implicit attention* (DIA), which is proposed in [30], leverages the inherent correlation of attention maps across layers by sharing a single self-attention module and employing an LSTM-based component to calibrate and connect these maps. Ioannides et al. proposed the multi-head *density adaptive attention mechanism* (DAAM) [31], a probabilistic attention framework with learnable mean and variance parameters, which enable it to dynamically model any distribution for feature recalibration. Building on the DAAM, the density adaptive transformer integrates this mechanism to enhance multi-modal information aggregation. Li et al. [32] introduced the Efficient Attention module guided by Normalization (EAN), which unifies feature normalization and attention mechanisms into a single, lightweight component.

In contrast to the above-mentioned non-probabilistic methods, some recent works [26,27,28] have proposed integrating probabilistic information into attention mechanisms. These approaches estimate the probability distribution of activation values within a CNN and use this information to guide attention. For example, attention weights may be derived based on the probability density function of the activations, allowing the model to focus on regions of the image that are less probable and therefore potentially more informative.

In summary, attention mechanisms have evolved from heuristic designs to more sophisticated designs (e.g., statistical principles). By leveraging channel, spatial, non-local, and self-attention, and probabilistic mechanisms, CNN models can potentially achieve improved performance across a wide range of computer vision tasks.

### 2.2. Contributions of Proposed Approach

It is important to highlight that the proposed attention mechanism differs from three conventional probabilistic methods [26,27,28] in the following two key aspects:

- Firstly, the proposed method constructs an attention map by estimating the probabilistic distribution of activation maps and correlating these with attention weights, providing a statistically grounded alternative to heuristic approaches. To be more specific, a Laplace distribution is used in this paper. This is very different from a Gaussian process [26], a Gaussian distribution [28], and an energy function [27]. An experiment was conducted by using the CIFAR-10 and CIFAR-100 datasets to justify the choice of the Laplace distribution by using five goodness-of-fit measures, as shown in Section 3.2.
- Secondly, unlike the channel design in [26] and the 3D attention map designs in [27,28], the proposed method applies a 2D attention design, making it computationally efficient and more straightforward to integrate with existing CNN layers.

## 3. Proposed Approach

### 3.1. Motivation

The proposed approach is driven by two key motivations. Firstly, it introduces a novel probabilistic attention mechanism that adaptively refines feature representations within a CNN model. The key idea behind this mechanism is to leverage statistical assumptions, more specifically, a Laplace distribution, to model the activation values at various layers of the network. Based on this distributional assumption, the proposed approach estimates the probability density function of these activation values. Once it is computed, it generates an attention map by applying a negative correlation mapping between the estimated probability distribution values and the attention weights for each pixel. This process allows the model to emphasize areas of lower probability (i.e., more informative or less common features) and suppress areas of higher probability, leading to a more refined feature representation. In essence, the attention mechanism dynamically adjusts the importance of each pixel based on its probabilistic significance within the learned distribution, enhancing the model’s ability to focus on the most relevant parts of the input.

Secondly, the proposed attention mechanism is designed as a plug-and-play module that can be seamlessly integrated into existing CNN architectures without significant modification. This plug-and-play nature offers two major benefits. First, it is compatible with a wide range of CNN models, allowing for performance improvements without the need to redesign the network. Second, the module is lightweight and computationally efficient, adding only little overhead in terms of memory and processing.

By combining a probabilistic attention mechanism with a plug-and-play integration strategy, the proposed approach provides a powerful tool for improving the performance of CNNs across various tasks while maintaining ease of integration and computational efficiency. Both of these two will be described in the following two sections in detail.

### 3.2. Probabilistic Distribution of Activations

To address the first issue of formulating the distribution of activations of CNN models, instead of applying the Gaussian distribution [28], this paper proposes to apply the Laplace distribution. This distribution is chosen because it models the concentration of information around a central value, which fits the context of feature map activations.

To justify the effectiveness of the proposed usage of the Laplace distribution, an experiment was conducted to fit the distribution of activations by comparing the Gaussian distribution and the Laplace distribution. For that, a goodness-of-fit measure was used to evaluate how well a statistical distribution fits a set of observed data (i.e., the activations of CNN models in this paper). It quantifies the discrepancy between the predicted values from the model and the actual observed values. Five goodness-of-fit measures were used in this paper. Considering the observed activations (denoted by *x*) of the CNN model and the predicted values (denoted by *y*) from the fitted distribution, these five measures are defined as follows [33]:

- *Deviation of Gain* (DG): It assesses the difference between the *cumulative distribution functions* (CDFs) of the observed data and the fitted distribution. It measures how the predicted distribution deviates from the observed distribution in terms of the gain as
  (1) $$DG=\sum_{i=1}^{n}\left(CDF_{\mathrm{obs}}\left(x_{i}\right)-CDF_{\mathrm{pred}}\left(y_{i}\right)\right),$$
  where $CDF_{\mathrm{obs}}\left(x_{i}\right)$ and $CDF_{\mathrm{pred}}\left(y_{i}\right)$ represent the cumulative distribution functions of the observed data and the fitted distribution, respectively.
- *Kling–Gupta Efficiency* (KGE): It combines three components, correlation, bias, and variability, to provide a balanced evaluation of model performance as
  (2) $$KGE=1-\sqrt{{\left(r-1\right)}^{2}+{\left(\alpha -1\right)}^{2}+{\left(\beta -1\right)}^{2}},$$
  where *r* is the Pearson correlation coefficient between *x* and *y*, $\alpha =\frac{\sigma_{y}}{\sigma_{x}}$ is the ratio of standard deviations, and $\beta =\frac{\mu_{y}}{\mu_{x}}$ is the bias ratio.
- *Mean Absolute Error* (MAE): It measures the average magnitude of errors between observed values and predicted values as
  (3) $$MAE=\frac{1}{n}\sum_{i=1}^{n}\left|x_{i}-y_{i}\right|,$$
  where $x_{i}$ represents the observed values and $y_{i}$ represents the fitted values.
- *Modified Nash–Sutcliffe Efficiency* (MNSE): It adjusts the traditional Nash–Sutcliffe Efficiency to account for bias and other systematic errors. It assesses the predictive accuracy relative to the variability of the observed data as
  (4) $$MNSE=1-\frac{\sum_{i=1}^{n}{\left(x_{i}-y_{i}\right)}^{2}}{\sum_{i=1}^{n}{\left(x_{i}-\bar{x}\right)}^{2}},$$
  where $\bar{x}$ is the mean of the observed values.
- *Ratio of standard deviations* (RSD): It compares the spread of the predicted values to the observed values as
  (5) $$RSD=\frac{\sigma_{y}}{\sigma_{x}},$$
  where $\sigma_{y}$ and $\sigma_{x}$ are the standard deviations of the predicted and observed data, respectively.

In this experiment, the CIFAR-10 and CIFAR-100 [34] datasets were used, where 100 images were randomly selected as test images. For each image, a ResNet-50 model pre-trained on the ImageNet dataset was analyzed. Feature values were extracted from the activation maps subsequent to the ReLU layers within all the bottleneck blocks of the residual branch. For each channel, the feature values were fitted into a Gaussian distribution and a Laplace distribution. Five goodness-of-fit measures were calculated and averaged over all images and all channels to obtain the performance comparison presented in Table 2. It should be noted that channels exhibiting a variance lower than $1\times 10^{-6}$ were systematically discarded from both distribution fittings due to their negligible statistical features and lack of contribution to the analysis. As seen in Table 2, the proposed Laplace distribution can fit the distribution of activations better by achieving better goodness-of-fit measurements.

### 3.3. Proposed Probabilistic Attention Function

The proposed probabilistic attention function can be obtained as follows.

First, the distribution of activations is formulated by using Laplace distribution as

(6) $$p\left(x\right)=\frac{\alpha }{2\lambda }\exp\left(-\frac{\left|x-\mu \right|}{\lambda }\right),$$

where μ and λ are two parameters of this distribution estimated from the activations and α is the additional trainable parameter. As seen in (6), the proposed attention mechanism involves three parameters. The two parameters μ and λ are derived from the probabilistic distribution of activations when integrated into the existing CNN model. They are not trainable and are calculated directly from the data. The parameter α is an additional trainable parameter introduced by the attention mechanism. When the attention mechanism is incorporated into the CNN backbone, the backbone is initialized by using a pre-trained CNN model. Subsequently, all trainable parameters, including α and those of the backbone, are optimized during the training process.

Then, an attention map is further constructed by applying a negative correlation mapping between the estimated probability distribution values and the attention weights for each pixel as

(7) $$m\left(x\right)=sigmoid\left(\frac{1}{p(x)+1}\right).$$

Finally, the attention is produced independently for each channel and applied to the original feature map by element-wise multiplication.

**Figure 1:**
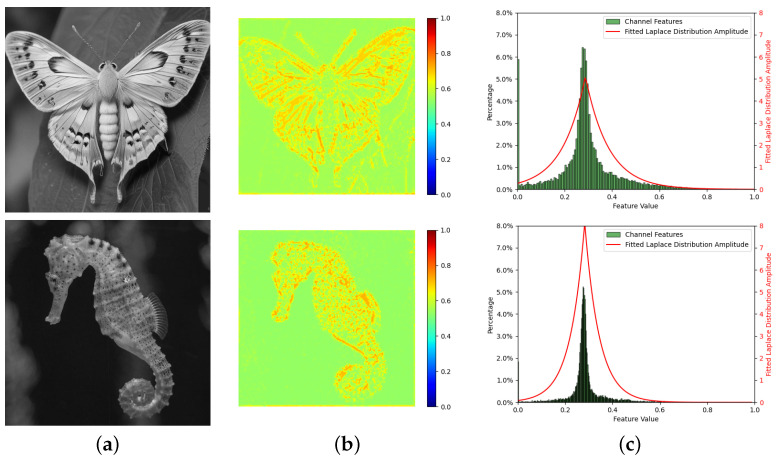
An illustration of the input image (**a**), its activation map (**b**), and the fitted Laplace distribution (red curve in (**c**)).

To illustrate the performance of the proposed probabilistic attention function, Figure 1 presents the input image, its associated activation map from a pre-trained ResNet-50 model, the fitted Laplace distribution, and the resulting probabilistic attention map. As seen in Figure 1, the attention adjusted by the Laplace distribution effectively highlights the important (more meaningful) regions of the input image.

### 3.4. Integration Strategy

**Figure 2:**
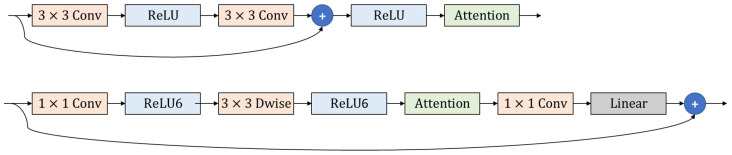
An illustration of how the proposed plug-and-play attention mechanism (green box) can be integrated into the residual block used in the ResNet model [35] (**top image**) or the inverted residual block used in the MovileNetV2 model [36] (**bottom image**).

The proposed attention mechanism is designed as a plug-and-play module, making it easy to seamlessly integrate into a wide range of existing CNN architectures without requiring major modifications. There are two typical scenarios for such integration. For CNN models with skip connections, such as ResNet [35], they integrate the proposed attention mechanism after the ReLU layer of each residual block. This ensures that the module refines the feature maps at a critical point in the network, enhancing its ability to capture important features. On the other hand, for CNN models with inverted residual blocks, such as the MobileNetV2 [36], the proposed module is inserted after the last ReLU layer. These two examples are illustrated in Figure 2.

### 3.5. Summary of Proposed Approach

The proposed probabilistic attention mechanism consists of a calculation of the probabilistic attention map and its integration with an existing CNN model. The details of the proposed approach are summarized as follows.

First, the activation values are formulated with a Laplace distribution (6). Feature maps of CNN models consist of activation values corresponding to different regions in the input image. The Laplace distribution estimated from the activations essentially assigns a probability value to each activation, representing how important that activation value is.

Next, the probabilistic attention map is calculated. A negative correlation mapping is applied between the probability density values and the attention weights (7), following the intuition that those with lower probability under the Laplace distribution are often more informative. Consequently, these regions should receive higher attention weights, while more common activations (those with higher probabilities) receive lower weights.

Then, the generated attention map is applied to the original feature map by element-wise multiplication. This step adjusts the feature representation by amplifying the most informative (less common) regions of the image and reducing the emphasis on less relevant (more common) regions. The probabilistic attention map is designed to be a plug-and-play module, which means that it can be integrated into any existing CNN architecture, as shown in Figure 2.

In summary, the proposed probabilistic attention map enhances feature representation learning and can be easily integrated into various CNN architectures for a wide range of computer vision tasks.

## 4. Experimental Results

### 4.1. Experimental Setup

The proposed probabilistic attention mechanism was evaluated in the context of an image classification task. The CIFAR-10 and CIFAR-100 datasets were selected [34] in the experiments, as they are well-established benchmarks in computer vision research. These datasets provide a rich variety of complex images, making them essential to assessing model performance. CIFAR-10 includes 60,000 32×32 color images spread across 10 distinct classes, with each class containing 6000 images. Of these, 50,000 images were allocated for training, while 10,000 were reserved for testing. CIFAR-100, in contrast, introduces greater complexity by offering 100 classes with 600 images per class.

### 4.2. Baseline Models

Two baseline CNN models were selected as vision backbones, including the ResNet series [35] and MobileNetV2 [36], both of which are widely used in CNN-based architectures. ResNet is commonly employed as the visual encoder in smaller-scale foundation models due to its effective use of residual connections, while MobileNetV2, with its inverted residual block structure, is popular as a visual encoder in lightweight models. These two architectures serve as strong baselines for evaluating the effectiveness of the proposed attention module.

Four probabilistic attention methods [13,22,27,28] were selected to be compared with the proposed approach. These three attention mechanisms were integrated into the CNN backbone models to be evaluated in the image classification task.

### 4.3. Performance Metrics

Two performance metrics were used to evaluate the image classification performance of the baseline CNN models with the integration of various attention modules. Top-1 accuracy refers to the percentage of times the model’s highest probability prediction matches the correct label in an image classification task. It evaluates the model’s ability to predict the exact class with its top prediction. On the other hand, top-5 accuracy measures how often the correct label is among the model’s top five predicted classes.

### 4.4. Implementation Details

The implementation details of the image classification models are described as follows. The ResNet models were trained by using zero-padding shortcuts to increase dimensions in all residual blocks, with all shortcuts being parameter-free. For the implementation of MobileNetV2, the stride of the first convolutional layer and the second inverted residual block were modified from 2 to 1, compared with the configuration described in [36]. Training was performed on a 4090 laptop GPU with a mini-batch size of 128, utilizing *stochastic gradient descent* (SGD) with a weight decay of 0.0001 and a momentum of 0.9, Kaiming Normal initialization, and batch normalization, without applying dropout. Specifically, for both ResNet and MobileNetV2, the initial learning rate was set to 0.1, which was reduced by a factor of 10 at 32k and 48k iterations, with training concluding at 64k iterations. In addition, for the ResNet-110 model, a warm-up phase was applied, where the learning rate was 0.01 until the training error dropped below 80%. The implementation of the proposed approach is available at https://github.com/ownEyes/Sensors-PAM-Laplace (accessed on 20 December 2024).

### 4.5. Performance Comparison

**Figure 3:**
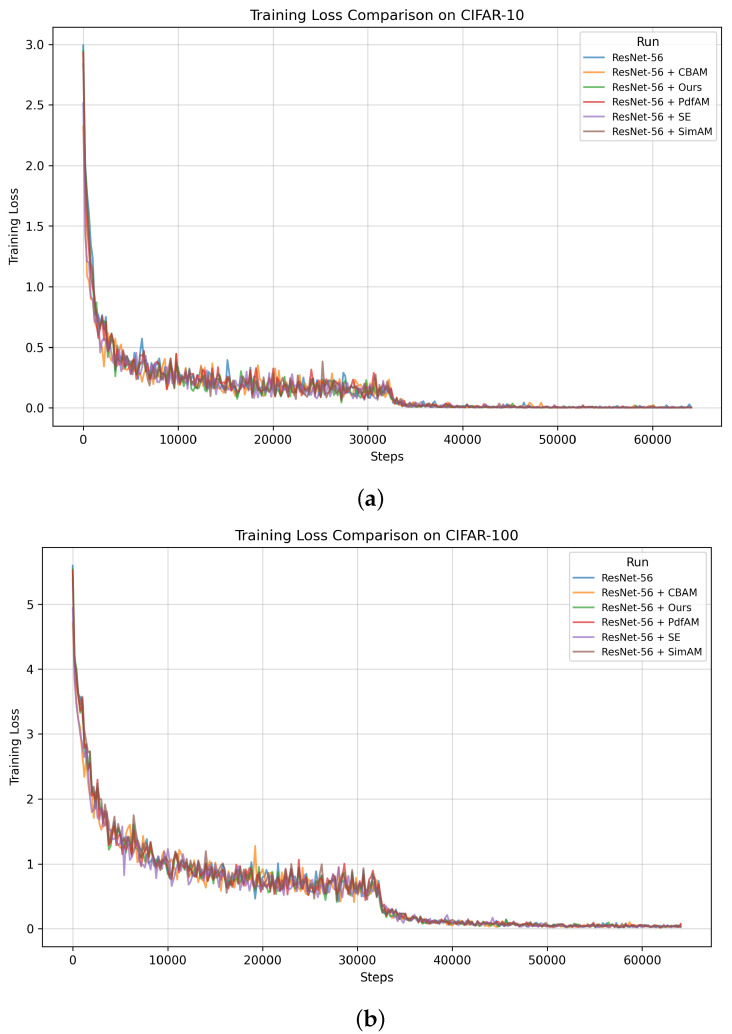
The model training loss curves of various methods using the CIFAR-10 dataset (**a**) and the CIFAR-100 dataset (**b**).

**Figure 4:**
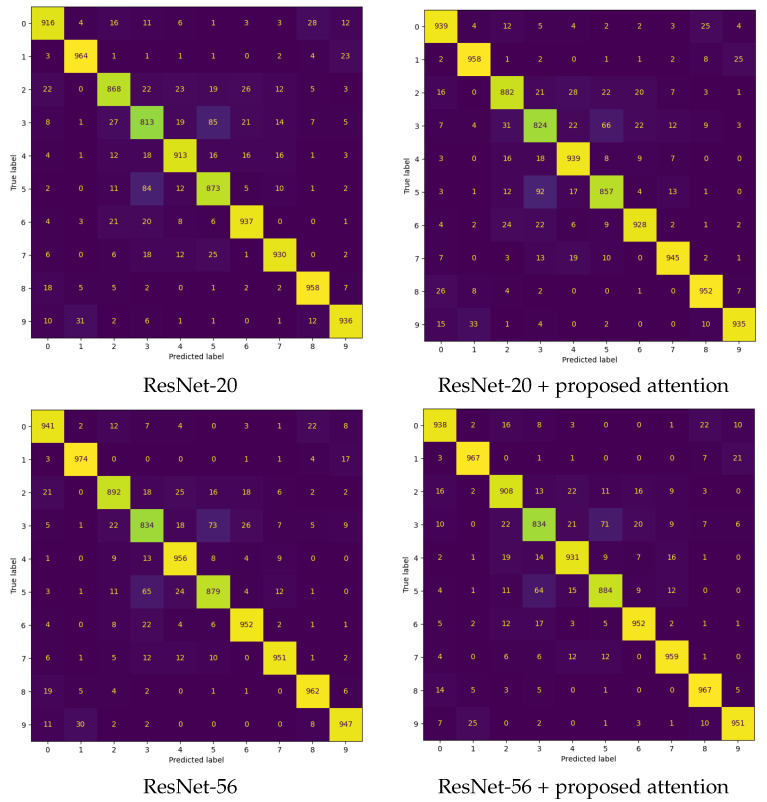
The confusion matrices for various backbones + the proposed attention mechanism on the CIFAR-10 dataset.

**Figure 5:**
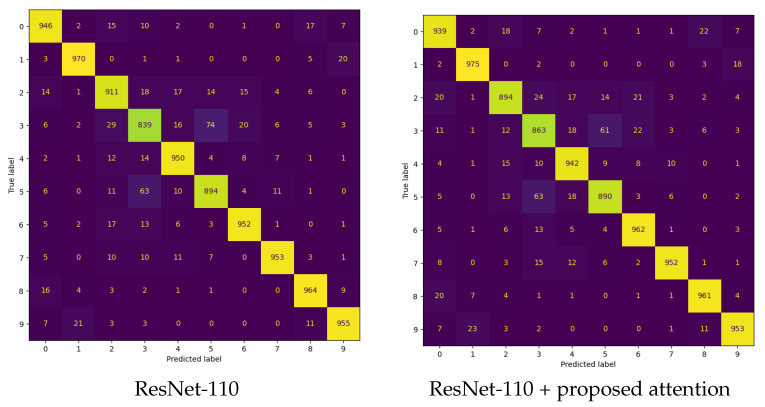

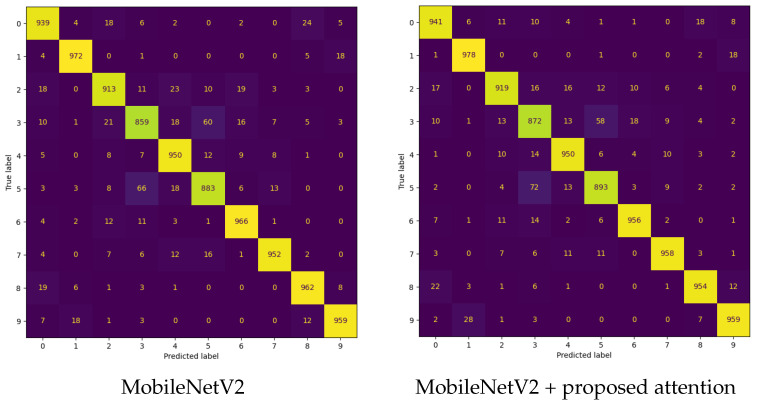
The confusion matrices for various backbones + the proposed attention mechanism on the CIFAR-10 dataset.

The first experiment was aimed to assess how well the proposed attention module enhances the image classification performance of CNN backbone models. Table 3 and Table 4 present the image classification performance (Top-1 Acc., Top-5 Acc., Prec.@Top-1, Rec.@Top-1, and F1@Top-1) for various baseline CNN architectures (i.e., ResNet-20, ResNet-56, ResNet-110, and MobileNetV2) integrated with five different attention modules (i.e., [13,22,27,28] and the proposed probabilistic attention mechanism). The experiments were conducted on the CIFAR-10 and CIFAR-100 datasets, and the results were averaged over five trials, with performance reported in the mean±std format. The training loss curves are provided in Figure 3. Figure 4 and Figure 5 present the confusion matrices of various backbones with the incorporation of the proposed attention mechanism on the CIFAR-10 dataset. Table 5 compares their computational complexity.

As seen in Table 3, for the CIFAR-10 dataset, across all CNN backbones, the proposed attention mechanism outperformed the baseline and the other two attention modules in terms of both top-1 and top-5 accuracy in many experiments. The improvement is particularly notable in the ResNet-20 model, where the proposed method achieved the highest top-1 accuracy of 91.62%. For the CIFAR-100 dataset, in Table 4, similar trends were observed. The proposed method outperformed the baseline and competing attention modules in most cases. In the ResNet-110 model, the proposed attention module also consistently yielded the best performance, with the highest top-1 accuracy of 71.65%.

Overall, Table 3 and Table 4 show that integrating attention modules into baseline neural networks improved classification accuracy on both CIFAR-10 and CIFAR-100. Notably, the proposed method frequently achieved the highest top-1 and top-5 accuracy, showing that its design can extract more informative features than the baseline and previously published attention mechanisms. The improvements are consistent, suggesting that these methods enhance the network’s representational capabilities. While the gains on CIFAR-10 are somewhat smaller, the enhancements on CIFAR-100 are often more significant, reflecting the method’s particular strength in handling more complex classification tasks. Overall, the experimental results shown in these tables verify that the proposed probabilistic attention mechanism effectively enhances image classification performance across multiple CNN backbone architectures and image datasets.

### 4.6. Ablation Study

The second experiment was to conduct an ablation study to evaluate the parameters used in the proposed attention mechanism (6), which could potentially introduce three scaling parameters (α,β,γ) as

(8) $$p\left(x\right)=\beta +\frac{\alpha }{2\lambda }\exp\left(-\frac{\gamma |x-\mu |}{\lambda }\right),$$

where μ and λ are two parameters of this distribution estimated from the activations and α, β, and γ are the additional trainable parameters. After that, the attention weights are calculated via $m\left(x\right)=\frac{1}{p(x)+1}$. To evaluate the contributions of these three parameters, an ablation study was conducted by using the ResNet-20 backbone CNN model on the CIFAR-10 and CIFAR-100 datasets. The ResNet-20 backbone model was integrated with the proposed probabilistic attention module with various configurations of scaling parameters defined in (8). The top-1 image classification accuracy is reported in Table 6, from which one can see that the proposed attention mechanism with only the parameter α achieved the best image classification performance.

## 5. Conclusions

This paper has proposed a new probabilistic attention mechanism by estimating the probabilistic distribution of activation maps and correlating attention weights with the probability density function values. The probabilistic attention map is applied to the feature maps via element-wise multiplication. In addition, the proposed attention module is integrated into existing CNNs as a plug-and-play component, enhancing image classification performance. As verified in the experimental results, the proposed probabilistic attention mechanism can effectively improve the image classification performance across many vision backbone models.

The probabilistic attention mechanism proposed in this paper holds significant promise for various sensing and imaging applications due to its plug-and-play nature. A key direction for future research is to extend its application beyond image classification to tasks like video classification. Video classification presents unique challenges, such as handling dynamic spatial and temporal data and distinguishing relevant frames or regions in dynamic scenes. By incorporating the proposed mechanism, vision models can prioritize critical features, such as target movements, while reducing focus on less relevant areas. Another promising avenue is exploring the integration of the attention module with diverse vision backbones, particularly transformer-based models. Given their architectural differences from CNNs, it is a valuable research direction to study how the probabilistic attention mechanism enhances transformer models.

## Figures and Tables

**Table 1:** An overview of attention mechanisms in vision-based sensing and imaging systems.

Method	Year	Attention Design	Is It a Probabilistic Method?
[13]	2018	Channel	-
[14]	2019	Channel	-
[15]	2020	Channel	-
[16]	2020	Channel	-
[17]	2023	Channel	-
[18]	2022	Non-local	-
[19]	2023	Self	-
[20]	2015	Spatial	-
[21]	2018	Spatial	-
[22]	2018	Spatial and channel	-
[23]	2019	Spatial and channel	-
[24]	2020	Spatial and channel	-
[25]	2022	Spatial and channel	-
[26]	2022	Channel	*√*
[27]	2021	Spatial	*√*
[28]	2023	Spatial	*√*
Proposed approach	-	Spatial	*√*

**Table 2:** The performance evaluation of the proposed Laplace distribution using five goodness-of-fit measures [33] based on the CIFAR-10 and CIFAR-100 [34] datasets.

	Indication of	CIFAR-10 Dataset		CIFAR-100 Dataset	
**Measure**	**Better Performance**	**Gaussian [28]**	**Laplace**	**Gaussian [28]**	**Laplace**
DG	A smaller value	−0.912	−0.920	−0.912	−0.919
KGE	A value closer to 1	−0.025	−0.017	−0.026	−0.017
MAE	A smaller value	0.079	0.077	0.079	0.077
MNSE	A value closer to 1	−0.591	−0.551	−0.591	−0.550
RSD	A value closer to 1	0.951	0.955	0.951	0.955

**Table 3:** The image classification performance using the CIFAR-10 image dataset [34], including Top-1 Acc., Top-5 Acc., Prec.@Top-1, Rec.@Top-1, and F1@Top-1, for various baseline networks integrated with attention modules. All results are reported as mean±std by calculating the performance of each model over 5 trials. The best performance is presented in the bold format.

Attention	ResNet-20				
Module	Top-1 Acc.	Top-5 Acc.	Prec.@Top-1	Rec.@Top-1	F1@Top-1
Baseline	91.19 ± 0.18	99.71 ± 0.03	91.17 ± 0.22	91.18 ± 0.22	91.17 ± 0.22
+ [13]	91.60 ± 0.10	99.74 ± 0.04	**91.59 ± 0.14**	**91.60 ± 0.14**	**91.59 ± 0.14**
+ [22]	91.55 ± 0.16	**99.74 ± 0.03**	91.51 ± 0.19	91.50 ± 0.18	91.50 ± 0.18
+ [27]	91.52 ± 0.13	99.70 ± 0.03	91.51 ± 0.13	91.52 ± 0.13	91.50 ± 0.13
+ [28]	91.38 ± 0.16	99.73 ± 0.04	91.28 ± 0.25	91.30 ± 0.25	91.28 ± 0.25
+ Proposed	**91.62 ± 0.16**	99.71 ± 0.04	91.46 ± 0.15	91.48 ± 0.15	91.46 ± 0.15
	ResNet-56				
	Top-1 Acc.	Top-5 Acc.	Prec.@Top-1	Rec.@Top-1	F1@Top-1
Baseline	92.64 ± 0.30	99.75 ± 0.03	92.63 ± 0.35	92.63 ± 0.35	92.62 ± 0.35
+ [13]	**93.18 ± 0.14**	99.75 ± 0.04	**93.12 ± 0.21**	**93.13 ± 0.22**	**93.12 ± 0.22**
+ [22]	93.03 ± 0.14	**99.77 ± 0.05**	93.00 ± 0.19	93.01 ± 0.18	93.00 ± 0.18
+ [27]	92.89 ± 0.17	99.74 ± 0.02	92.85 ± 0.22	92.86 ± 0.21	92.85 ± 0.21
+ [28]	93.00 ± 0.12	99.73 ± 0.04	92.96 ± 0.14	92.97 ± 0.13	92.96 ± 0.13
+ Proposed	92.85 ± 0.10	99.75 ± 0.05	92.85 ± 0.06	92.87 ± 0.07	92.86 ± 0.06
	ResNet-110				
	Top-1 Acc.	Top-5 Acc.	Prec.@Top-1	Rec.@Top-1	F1@Top-1
Baseline	93.13 ± 0.15	99.75 ± 0.03	93.04 ± 0.23	93.05 ± 0.24	93.04 ± 0.24
+ [13]	**93.54 ± 0.20**	99.78 ± 0.03	**93.46 ± 0.21**	**93.46 ± 0.21**	**93.45 ± 0.21**
+ [22]	93.41 ± 0.18	**99.79 ± 0.04**	93.32 ± 0.24	93.33 ± 0.23	93.31 ± 0.23
+ [27]	93.33 ± 0.20	99.78 ± 0.03	93.36 ± 0.22	93.37 ± 0.21	93.36 ± 0.22
+ [28]	93.45 ± 0.12	99.75 ± 0.04	93.39 ± 0.28	93.40 ± 0.28	93.39 ± 0.29
+ Proposed	93.39 ± 0.13	99.77 ± 0.03	93.34 ± 0.09	93.34 ± 0.08	93.33 ± 0.08
	MobileNetV2				
	Top-1 Acc.	Top-5 Acc.	Prec.@Top-1	Rec.@Top-1	F1@Top-1
Baseline	**93.82 ± 0.13**	**99.82 ± 0.03**	93.71 ± 0.14	93.71 ± 0.13	93.70 ± 0.13
+ [13]	93.56 ± 0.19	99.79 ± 0.03	93.60 ± 0.24	93.58 ± 0.24	93.56 ± 0.24
+ [22]	93.42 ± 0.16	99.77 ± 0.03	93.35 ± 0.19	93.36 ± 0.18	93.35 ± 0.19
+ [27]	93.80 ± 0.11	99.75 ± 0.04	**93.71 ± 0.13**	93.71 ± 0.12	93.70 ± 0.12
+ [28]	93.61 ± 0.19	99.79 ± 0.04	93.61 ± 0.19	93.60 ± 0.20	93.60 ± 0.19
+ Proposed	93.71 ± 0.16	99.80 ± 0.04	93.71 ± 0.18	**93.72 ± 0.18**	**93.71 ± 0.18**

**Table 4:** The image classification performance using the CIFAR-100 image dataset [34], including Top-1 Acc., Top-5 Acc., Prec.@Top-1, Rec.@Top-1, and F1@Top-1, for various baseline networks integrated with attention modules. All results are reported as mean±std by calculating the performance of each model over 5 trials. The best performance is presented in the bold format.

Attention	ResNet-20				
Module	Top-1 Acc.	Top-5 Acc.	Prec.@Top-1	Rec.@Top-1	F1@Top-1
Baseline	66.07 ± 0.41	89.84 ± 0.23	66.84 ± 0.34	66.47 ± 0.29	66.50 ± 0.30
+ [13]	**67.04 ± 0.10**	90.16 ± 0.22	**67.50 ± 0.34**	**67.13 ± 0.22**	**67.13 ± 0.22**
+ [22]	66.79 ± 0.25	90.31 ± 0.17	67.45 ± 0.40	66.97 ± 0.15	66.98 ± 0.24
+ [27]	66.83 ± 0.44	90.00 ± 0.28	67.48 ± 0.37	66.98 ± 0.35	67.03 ± 0.36
+ [28]	66.29 ± 0.12	89.75 ± 0.13	66.95 ± 0.48	66.45 ± 0.32	66.49 ± 0.38
+ Proposed	66.86 ± 0.12	**90.22 ± 0.19**	67.35 ± 0.21	66.94 ± 0.20	66.97 ± 0.21
		ResNet-56			
	Top-1 Acc.	Top-5 Acc.	Prec.@Top-1	Rec.@Top-1	F1@Top-1
Baseline	69.41 ± 0.54	90.66 ± 0.13	69.95 ± 0.65	69.56 ± 0.65	69.59 ± 0.65
+ [13]	**70.32 ± 0.35**	**91.35 ± 0.11**	**70.98 ± 0.32**	**70.59 ± 0.25**	**70.63 ± 0.26**
+ [22]	70.20 ± 0.26	91.15 ± 0.15	70.64 ± 0.51	70.23 ± 0.46	70.26 ± 0.45
+ [27]	69.62 ± 0.47	90.84 ± 0.25	70.37 ± 0.78	69.87 ± 0.75	69.95 ± 0.76
+ [28]	69.67 ± 0.20	91.09 ± 0.32	70.24 ± 0.28	69.88 ± 0.36	69.91 ± 0.33
+ Proposed	69.70 ± 0.28	91.05 ± 0.16	70.55 ± 0.30	70.11 ± 0.30	70.16 ± 0.26
	ResNet-110				
	Top-1 Acc.	Top-5 Acc.	Prec.@Top-1	Rec.@Top-1	F1@Top-1
Baseline	71.29 ± 0.46	91.42 ± 0.16	71.54 ± 0.51	71.31 ± 0.53	71.29 ± 0.54
+ [13]	**72.11 ± 0.09**	**91.77 ± 0.21**	**72.39 ± 0.21**	**72.08 ± 0.22**	**72.07 ± 0.19**
+ [22]	71.80 ± 0.17	91.76 ± 0.23	72.01 ± 0.25	71.72 ± 0.21	71.72 ± 0.23
+ [27]	71.49 ± 0.39	91.56 ± 0.15	71.87 ± 0.31	71.56 ± 0.24	71.56 ± 0.26
+ [28]	71.26 ± 0.16	91.73 ± 0.17	71.58 ± 0.27	71.26 ± 0.26	71.28 ± 0.26
+ Proposed	71.62 ± 0.34	91.65 ± 0.12	71.98 ± 0.49	71.67 ± 0.43	71.68 ± 0.44
	MobileNetV2				
	Top-1 Acc.	Top-5 Acc.	Prec.@Top-1	Rec.@Top-1	F1@Top-1
Baseline	73.97 ± 0.31	92.97 ± 0.14	74.15 ± 0.34	73.97 ± 0.35	73.93 ± 0.34
+ [13]	73.78 ± 0.14	92.78 ± 0.11	73.85 ± 0.27	73.80 ± 0.24	73.71 ± 0.26
+ [22]	73.09 ± 0.19	92.33 ± 0.17	73.15 ± 0.31	73.09 ± 0.29	72.99 ± 0.30
+ [27]	74.21 ± 0.25	93.23 ± 0.05	74.44 ± 0.43	74.19 ± 0.41	74.19 ± 0.42
+ [28]	74.26 ± 0.29	93.05 ± 0.20	74.46 ± 0.33	74.23 ± 0.39	74.22 ± 0.37
+ Proposed	**74.42 ± 0.16**	**93.24 ± 0.14**	**74.65 ± 0.29**	**74.43 ± 0.29**	**74.41 ± 0.30**

**Table 5:** The computational complexity performance comparison for various baseline networks integrated with attention modules.

Module	FLOPs	# Parameters	FLOPs	# Parameters
	CIFAR-10 image dataset			
	ResNet-20		ResNet-56	
Baseline	41.31 M	0.270 M	127.62 M	0.853 M
+ [13]	41.49 M	0.271 M	128.15 M	0.859 M
+ [22]	41.89 M	0.272 M	129.36 M	0.863 M
+ [27]	41.31 M	0.270 M	127.62 M	0.853 M
+ [28]	41.31 M	0.271 M	127.62 M	0.856 M
+ Proposed	41.31 M	0.271 M	127.62 M	0.856 M
	ResNet-110		MobileNetV2	
Baseline	257.09 M	1.728 M	92.78 M	2.237 M
+ [13]	258.13 M	1.740 M	93.08 M	2.265 M
+ [22]	260.57 M	1.748 M	93.55 M	2.268 M
+ [27]	257.09 M	1.728 M	92.78 M	2.237 M
+ [28]	257.09 M	1.734 M	92.78 M	2.258 M
+ Proposed	257.09 M	1.734 M	92.78 M	2.258 M
	CIFAR-100 image dataset			
	ResNet-20		ResNet-56	
Baseline	41.32 M	0.276 M	127.63 M	0.859 M
+ [13]	41.49 M	0.277 M	128.15 M	0.865 M
+ [22]	41.90 M	0.278 M	129.37 M	0.869 M
+ [27]	41.32 M	0.276 M	127.63 M	0.859 M
+ [28]	41.32 M	0.277 M	127.63 M	0.862 M
+ Proposed	41.32 M	0.277 M	127.63 M	0.862 M
	ResNet-110		MobileNetV2	
Baseline	257.10 M	1.734 M	92.89 M	2.352 M
+ [13]	258.14 M	1.746 M	93.20 M	2.380 M
+ [22]	260.58 M	1.753 M	93.67 M	2.384 M
+ [27]	257.10 M	1.734 M	92.89 M	2.352 M
+ [28]	257.10 M	1.740 M	92.89 M	2.373 M
+ Proposed	257.10 M	1.740 M	92.89 M	2.373 M

**Table 6:** The ablation study of the options of three scaling parameters, α, β, and γ in (8), using top-1 accuracy image classification performance with a ResNet-20 backbone model. All results are reported mean±std by calculating the performance of each option over 5 trials. The best performance is represented in the bold format.

α	β	γ	CIFAR-10	CIFAR-100
-	-	-	92.18 ± 0.22	68.21 ± 0.12
*√*	-	-	**92.29 ± 0.11**	**68.46 ± 0.11**
-	*√*	-	92.02 ± 0.10	67.98 ± 0.29
-	-	*√*	92.23 ± 0.06	68.32 ± 0.26
*√*	*√*	-	92.07 ± 0.14	68.17 ± 0.37
*√*	-	*√*	92.26 ± 0.23	68.37 ± 0.44
-	*√*	*√*	92.25 ± 0.14	68.34 ± 0.17
*√*	*√*	*√*	92.22 ± 0.26	68.25 ± 0.29

## Data Availability

Data are contained within the article. These data were derived from the following resources available in the public domain: https://www.cs.toronto.edu/~kriz/cifar.html (accessed on 20 December 2024).
